# Unilateral Autoimmune Encephalitis: A Case Report on a Rare Manifestation of Myelin Oligodendrocyte Glycoprotein Antibody Disease

**DOI:** 10.7759/cureus.34994

**Published:** 2023-02-14

**Authors:** Mohan V. Sumedha Maturu, Aravind Varma Datla, Prajwala Maturu, Vinay B Talla, Sibasankar Dalai

**Affiliations:** 1 Neurology, Medicover Hospitals, Visakhapatnam, IND; 2 Internal Medicine, Medicover Hospitals, Visakhapatnam, IND; 3 Psychiatry, Government Hospital for Mental Care, Visakhapatnam, IND; 4 Neurosurgery, Medicover Hospitals, Visakhapatnam, IND; 5 Interventional Neuroradiology, Medicover Hospitals, Visakhapatnam, IND

**Keywords:** intravenous immunoglobulins (ivig), intravenous methylprednisolone pulse, mogad, pediatric brain mri, epilepsia partialis continua (epc), mog antibody-associated disease, inflammatory demyelination, myelin oligodendrocyte glycoprotein (mog) antibodies, cortical encephalitis, flames

## Abstract

Myelin oligodendrocyte glycoprotein (MOG)-associated disease (MOGAD) is a rare, antibody-mediated inflammatory demyelinating disorder of the central nervous system (CNS) that has varying phenotypes. FLAIR (fluid-attenuated inversion recovery)-hyperintense Lesions in Anti-MOG-associated Encephalitis with Seizures (FLAMES) is a much rarer manifestation of cortical encephalitis encountered in MOGAD. We report a rare case of a nine-year-old girl who presented with a drop in her academic performance and right-sided Epilepsia partialis continua. Magnetic resonance imaging (MRI) of the brain detected evidence for unilateral (left) cortical encephalitis with peri-ictal juxtacortical edema. An electroencephalogram revealed a hemi-generalized poly spike and wave discharges in the left hemisphere, several of which correlated with myoclonic jerks. The cerebrospinal fluid (CSF) analysis was normal. Autoimmune workup resulted in a positive serum MOG-immunoglobulin G (IgG), which confirmed the diagnosis of FLAMES. The child showed an excellent clinical response to intravenous methylprednisolone and intravenous immunoglobulins therapy.

## Introduction

Myelin oligodendrocyte glycoprotein (MOG)-associated disease (MOGAD) is a rare antibody-mediated inflammatory demyelinating disorder of the central nervous system (CNS). Despite earlier studies describing MOGAD as similar to neuromyelitis optica spectrum disorders (NMOSD), recent evidence describes MOGAD as a distinct disease with unique immunological characteristics [1-3]. It manifests with varying phenotypes, predominantly optic neuritis, myelitis, and encephalitis [1,2].

Encephalitis is a relatively rare manifestation of MOGAD compared to optic neuritis and myelitis. Till recently, encephalitis in MOGAD was thought to involve subcortical structures similar to acute disseminated encephalomyelitis (ADEM). Recently, a distinct and much rarer manifestation of MOGAD, cortical encephalitis, was described which is FLAIR (Fluid attenuated inversion recovery)-hyperintense Lesions in Anti-MOG-associated Encephalitis with Seizures (FLAMES). It was first described by Ogawa et al. in 2017 as a specific clinico-radiological syndrome, separate from other anti-MOG antibody-associated inflammatory demyelinating disorders. It often has an indolent clinical course, particularly in children [3-5].

Seizures, headaches, fevers, and cortical symptoms referable to the FLAMES location are the most common clinical manifestations. A vast majority have two or more of the four above-mentioned findings simultaneously [2,5].

We report a rare case of a nine-year-old girl who presented with a drop in her academic performance and right-sided weakness of her upper and lower limbs and face. Early recognition and prompt treatment led to an exceptional outcome.

## Case presentation

A nine-year-old girl was brought to our neurology department by her parents with a 2.5-month history of a drop in scholastic performance. Initially, her scholastic decline and inattention were perceived to result from a behavioral issue by her teacher, and the parents were intimated. She was taken to a child psychiatrist for evaluation. The child’s psychiatric evaluation was normal and was advised close monitoring to seek any evolution of symptoms. Two weeks later, she was noted to have brief episodes of right upper and lower limb jerks with preserved consciousness and awareness. She was initially started on oral carbamazepine by a pediatrician, which resulted in a transient cessation of the jerks.

**Figure 1:**
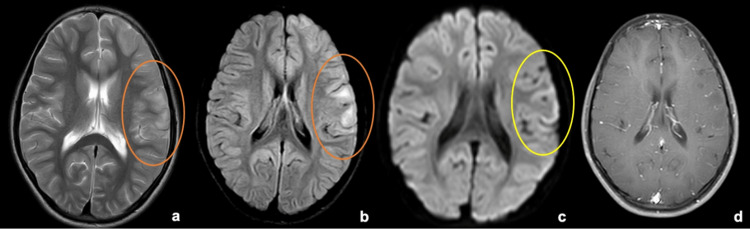
MRI brain at the upper section of the internal capsule From left to right: (a) T2W, (b) FLAIR, (c) DWI, (d) Post-contrast T1W images. Areas within the orange ellipse in (a) and (b) show focal T2/FLAIR hyperintensities in the left frontal cortical and juxta/subcortical regions with gyral swelling and sulcal effacement. The yellow ellipse marked in the DWI image (c) shows corresponding areas of cortical restriction. There is no significant post-contrast enhancement in this section (d). DWI - Diffusion-weighted imaging; FLAIR - Fluid attenuated inversion recovery; MRI - Magnetic resonance imaging; T1W - T1 weighted; T2W - T2 weighted

**Figure 2:**
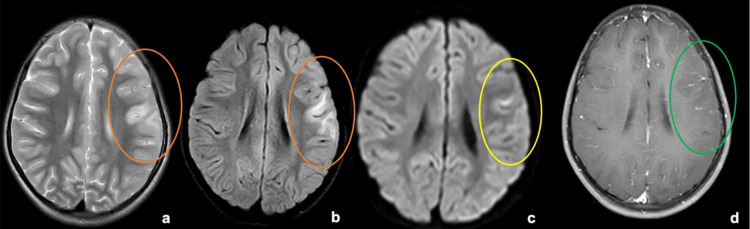
MRI brain sections above the level of the internal capsule From left to right: (a) T2W, (b) FLAIR, (c) DWI, (d) Post-contrast T1W images. Areas within the orange ellipse in (a) and (b) show focal T2/FLAIR hyperintensities in the left frontal cortical and juxta/subcortical regions with gyral swelling and sulcal effacement. The yellow ellipse marked in the DWI image (c) shows corresponding areas of cortical restriction. There is a thin leptomeningeal enhancement in the areas corresponding to sulcal effacement as marked with the green ellipse in (d). DWI - Diffusion-weighted imaging; FLAIR - Fluid attenuated inversion recovery; MRI - Magnetic resonance imaging; T1W - T1 weighted; T2W - T2 weighted

**Figure 3:**
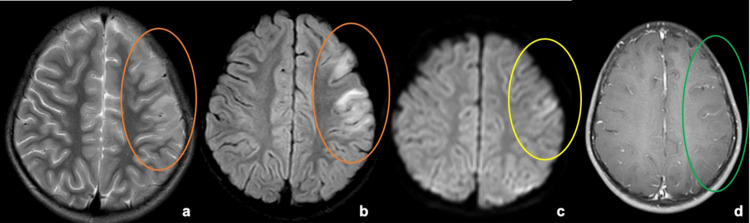
MRI brain sections at the level of the centrum semiovale From left to right: (a) T2W, (b) FLAIR, (c) DWI (d) Post-contrast T1W images. Areas within the orange ellipse in (a) and (b) show focal T2/FLAIR hyperintensities in the left frontal cortical and juxta/subcortical regions with gyral swelling and sulcal effacement. The yellow ellipse marked in the DWI image (c) shows corresponding areas of cortical restriction. There is a thin leptomeningeal enhancement in the areas corresponding to sulcal effacement as marked with the green ellipse in (d). DWI - Diffusion-weighted imaging; FLAIR - Fluid attenuated inversion recovery; MRI - Magnetic resonance imaging; T1W - T1 weighted; T2W - T2 weighted

In the last month, despite being on medication, the frequency increased to such a degree that the jerking was nearly continuous. The jerks also started affecting the right side of her face. This was associated with a drop in verbal output with intact comprehension and weakness of right-sided limbs with consequent walking difficulty. At this stage, the child was referred to our care. At the initial examination, the child was conscious, alert, and comprehending. She was having left hemispheric epilepsia partialis continua (EPC). Magnetic resonance imaging (MRI) of the brain detected evidence of unilateral (left) cortical encephalitis with peri-ictal juxtacortical edema (Figures 1-3).

An electroencephalogram revealed a hemi-generalized poly spike and wave discharges in the left hemisphere, several of which correlated with myoclonic jerks. Cerebrospinal fluid (CSF) demonstrated normal cells, proteins, and glucose. Initial possibilities of early Rasmussen encephalitis (prior to the development of atrophy) Vs. MOG-related unilateral encephalitis (FLAMES) were considered. Autoimmune workup for CSF N-methyl-D-aspartate receptor (NMDAR) antibody, serum NMDAR, voltage-gated potassium channel (VGKC), α-amino-3-hydroxy-5-methyl-4-isoxazolepropionic acid (AMPA) receptor, and gamma-aminobutyric acid (GABA) antibodies were negative.

Serum MOG cell-based indirect immunofluorescence assay was positive. Serum metabotropic glutamate receptor 3 (mGLUR3) was negative. The child was started on a five-day 30 mg/kg/day pulsed intravenous methylprednisolone (IVMP) along with 2 g/kg intravenous immunoglobulins (IVIg). She had a significant improvement in EPC and started walking independently. She was maintained on a monthly 1g/kg IVIg pulse. At her one-month follow-up, there were no neurological deficits. The patient was followed up for four months during which there was no recurrence of symptoms. She lost follow-up later on.

## Discussion

We report a case of a young girl with FLAMES, a novel clinco-radiographic entity. FLAMES is a subtype of the MOGAD spectrum.

In 2017, Ogawa et al. performed a retrospective cross-sectional study among a cohort of 24 consecutive adult patients with steroid-responsive encephalitis of unknown etiology after encountering an index case of MOG antibody-positive unilateral cortical encephalitis with epileptic seizure. Four MOG-IgG-positive patients were identified, all of whom had similar distinct unilateral cortical T2-FLAIR hyperintensities, which resolved with high-dose IVMP. In contrast, none of the MOG-IgG-negative patients showed neuroimaging findings suggesting anti-MOG-associated encephalitis [4]. Further studies into this phenomenon by Budhram et al. revealed this observation to be a distinct clinico-radiographic syndrome, which was labeled as FLAMES in 2019 [2].

The discovery of a disease-specific serum IgG antibody that selectively binds MOG has increased the understanding of the diverse spectrum of MOGAD. MOG is a member of the immunoglobulin superfamily and a lesser component of myelin. MOG is expressed on the surface of oligodendrocytes, on the outermost part of the myelin sheath. It is highly immunogenic, making it a potential target for MOG antibodies [6,7]. The function of MOG is yet to be fully understood. But it is hypothesized to act as a cell adhesion molecule, a regulator of microtubule stability, and a modulator of myelin immune interactions [6]. MOG can directly activate the classical pathway of the complement cascade by binding to the C1q and C3d components, which can activate the complement system. This potential role of MOG in the complement cascade may provide insight into the role of MOG in the demyelinating process [1,8]. A majority of human pathological studies on MOGAD are derived from brain lesions identified during autopsy or biopsy. These studies have revealed coexisting perivenous and confluent demyelination. A CD4+ T cell reaction with granulocytic inflammation is typical, which differs from MS, in which a CD8+ infiltrate predominates [9,10]. The overall pathology depicts an antibody-mediated central nervous system (CNS) demyelinating disease distinct from multiple sclerosis (MS).

The mean presenting age is 29 years (range 11-46 years), with a male predominance (60%) [11]. ADEM and other cerebral presentations are more common in the pediatric age groups. In children, the course is usually indolent and monophasic [1]. The characteristic clinical features include seizures (85%), headache (70%), fever (65%), and cortical symptoms referable to the FLAIR hyperintense regions (55%). However, 95% of the patients depict two or more of the above findings [2]. Our patient had both seizures and FLAIR hyperintense referable cortical symptoms and tested positive for serum anti-MOG antibody. Moreover, she had a decline in scholastic performance. Though not characteristic, similar findings were observed by Budhram et al., wherein 55% of their patients had a history of altered behavior or mental status at presentation [2].

In a few of the earlier reports, subcortical FLAIR hyperintensities on MRI were considered as an exclusion criterion. Later reports, however, placed little emphasis on these findings. In our case, MRI was done while the child was having EPCs and the juxtacortical FLAIR hyperintensities are likely because of peri-ictal edema, as there is no corresponding diffusion-weighted imaging (DWI) restriction suggesting that it is a vasogenic pathology. While the cortical gyral FLAIR hyperintensities showed corresponding DWI restriction suggesting a cytotoxic pathology secondary to encephalitis, there is also associated sulcal effacement and corresponding focal thin leptomeningeal enhancement, as is noted in a few of the earlier reports [3].

Cell-based assays with full-length human MOG as the target antigen are considered the gold standard method for detecting anti-MOG antibodies. Serum is a better sampling source than CSF, which has a lower concentration of antibodies [12]. The timing of the sample is vital to the detection of MOG-IgG. MOG-IgG levels fluctuate with disease activity. Higher median concentrations are observed during acute attacks and low levels during remission or in the chronic phase. They may even be absent after a monophasic incident [13]. Other methods like immunohistochemistry, peptide-based enzyme-linked immunosorbent assay (ELISA), or Western blotting are not typically recommended because of their low specificity [1].

The concept of MOGAD and FLAMES are relatively novel entries into the domain of the antibody-mediated inflammatory disorder spectrum. The low prevalence, varied clinical features, and age-related differences in clinical presentation all contribute to the sparsity of medical literature on this topic. To date, no large-scale, multicentric treatment trial has been conducted. As a result, there are no evidence-based guidelines. Since MOGAD/FLAMES have only recently been delineated from NMOSD, there is an overlap in the treatment approaches. The treatment guidelines are derived from expert opinions.

High-dose IVMP (30 mg/kg/day for 5-10 days) is the treatment modality of choice, particularly for acute exacerbations. For patients with steroid unresponsiveness or an aggressive clinical course, early initiation of IVIg or plasma exchange may be beneficial [1-5]. Though high-dose IVMP is effective in inducing remission, there is a substantial risk of relapse after steroid dose reduction or discontinuation [4,14,15]. Therefore, maintenance therapy is critical in anti-MOG antibody-positive cases. A protracted duration (oral prednisolone 10 mg over three months), and gradual tapering off oral corticosteroids has nearly halved the relapse rate (from 47% to 25%) [13,16].

Glatiramer acetate, natalizumab, and interferon beta, which are classically used as disease-modifying drugs in MS, have been ineffective or worsened the disease [13,17].

Immunosuppressants such as azathioprine, methotrexate, mycophenolate mofetil, rituximab, and IVIg (repeated cycles) have been studied to combat the potentially hazardous consequences of long-term steroid therapy. A recent retrospective multicentric study reported that IVIg infusions demonstrated the most promising results (relapse rate of ≤ 20%) compared to other agents (relapse rate 59-74%). Hence, we opted for monthly pulse IVIg therapy [1,18].

## Conclusions

This case report adds to the literature evidence for the recently described FLAMES clinico-radiographic entity. Though previously considered a variant of NMOSD, MOGAD is now recognized as a separate, antibody-mediated inflammatory demyelinating pathology. The discovery of novel antibodies has redefined the thought process about inflammatory demyelinating CNS disorders. The initial clinical presentation may be varied, which can add to the diagnostic difficulty. Hence, physicians should remain cognizant of this. Physicians should notice FLAMES as a unique sub-entity of the MOGAD spectrum with distinctive clinical and radiological findings and could be a differential diagnosis for several focal encephalitis-like presentations. The method and timing of testing for MOG antibodies are crucial. Cell-based assays have the highest sensitivity and are considered the gold standard. Repeat testing might be beneficial even after initial negative results if the clinical suspicion is strong. Early recognition and prompt treatment are crucial for achieving the best possible outcomes.
